# Hemodynamic and ventilatory changes in pediatric patients with special needs: A comparative clinical study

**DOI:** 10.4317/jced.59951

**Published:** 2022-11-01

**Authors:** Silvia Pérez-García, Juan-Antonio Ruiz-Roca, Cristobal Añez, Pía López-Jornet, Jordi Gargallo-Albiol

**Affiliations:** 1 DDS, MS. Assistant Professor. Department of Oral and Maxillofacial Surgery. Universitat Internacional de Catalunya, Sant Cugat del Vallés (Barcelona), Spain; 2 DDS, MS, PhD. Assistant Professor. Department of Stomatology. Faculty of Dentistry. University of Murcia, Murcia, Spain; 3 MD, PhD. Associate Professor of Anesthesia. Universitat Rovira i Virgili. Anesthetist, Hospital Universitari de Tarragona Joan XXIII, Tarragona, Spain; 4 MD, DDS, MS, PhD. Chairman and Professor of Oral Medicine. Department of Stomatology. Faculty of Dentistry. University of Murcia, Murcia, Spain; 5 DDS, MS, PhD. Associate Professor. Department of Oral and Maxillofacial Surgery. Universitat Internacional de Catalunya, Sant Cugat del Vallés (Barcelona), Spain. Adjunct Clinical Professor. Department of Periodontics and Oral Medicine, University of Michigan School of Dentistry, Ann Arbor, MI, USA

## Abstract

**Background:**

Very limited data are available on the hemodynamic and ventilatory changes during sedation and general anesthesia using bispectral index (BIS) monitoring in intellectually disabled children. The purpose was to determine the hemodynamic and ventilatory changes after propofol and sevoflurane administration in children with special needs (CSN) versus healthy children (HC) during dental treatment.

**Material and Methods:**

Forty pediatric patients needing dental treatment were allocated into two groups: children without systemic disease (healthy children [HC]) and mentally disabled children (children with special needs [CSN]). Sevoflurane in oxygen (100% oxygen, 5 l/min) and continuous propofol infusion (target-controlled infusion [TCI], 2 µg/ml) were used as sedative agents, and 2% lidocaine with 1:80,000 adrenaline was used as local anesthesia in both groups. Heart rate (HR), oxygen saturation (SaO2), respiratory rate (RR), exhaled carbon dioxide (CO2), blood pressure (BP) and bispectral monitoring (BIS) values were recorded during the entire dental treatment procedure.

**Results:**

A statistically significant decrease in systolic BP, diastolic BP and RR was observed, with no significant differences between the healthy and disabled groups. In contrast, the HR and BIS values were lower in the CSN group than in the healthy patients (*p* ≤ 0.05).

**Conclusions:**

Patients with special needs had lower HR and BIS values than healthy patients, while BP, SaO2 and exhaled CO2 showed similar results in both groups.

** Key words:**Bispectral index, hemodynamic changes, ventilatory changes, pediatric patients, special needs.

## Introduction

Providing dental care for anxious and fearful patients is a common major challenge for dentists (1,2). Moreover, in the case of pediatric patients, the dental visit can represent a shocking event (3). When an anxiety situation is combined with the administration of local anesthetics and vaso-constrictors, the undesirable effects upon the cardiovascular system, with the secretion of endogenous catecholamines, may be incremented (4). Patients under three years of age or with special needs require additional support beyond local anesthesia in order to receive dental treatment (3,5). The traditional non-pharmacological management strategies are unable to resolve resistive and uncooperative behavior in these cases (3), and deep sedation or general anesthesia is required in order to allow satisfactory dental treatment to be carried out (6).

While conscious and moderate sedation is often sufficient for performing the majority of dental treatment in adults, deeper sedation levels or general anesthesia may occasionally be required in children under 7 years of age and in patients with intellectual disorders (3,6-8).

Children with special needs are defined by the presence of physical, developmental, mental, sensory, behavioral, cognitive or emotional disabilities that require differentiated medical treatment, special medical interventions, and/or the use of specialized services or programs (9,10). In children with special needs, there may be criteria justifying more frequent use of general anesthesia for dental treatment. These criteria include the need for extensive treatment and bad behavior and, to a lesser extent, possible associated medical problems (8).

In accordance with the American Academy of Pediatric Dentistry and the American Society of Anesthesiologists (ASA), patients under deep sedation and general anesthesia must be monitored continuously by the anesthesiologist. It is mandatory to monitor oxygenation through pulsioximetry, ventilation through the end-tidal carbon dioxide (EtCO2) concentration using capnography and respiratory rate, and hemodynamics though heart rate and blood pressure (4,6,11-14) - all of which shall be recorded at least every 5 minutes. (6) An appropriately trained person must continuously observe the patient until discharge (6).

Apart from basic monitoring, accurate assessment of the depth of sedation, or the performance of general anesthesia, a tool that is reliable and easy to use in the clinical setting, such as the bispectral index (BIS), provides interesting information (15). Bispectral index monitoring is a noninvasive technique used in clinical practice to evaluate the level of hypnosis (3,11,15), and was approved by the United States Food and Drug Administration (FDA) in 1996 for monitoring the level of hypnosis in clinical practice (16). This index is based on the principle that the electroencephalogram (EEG) waveforms change with the level of alertness of the patient (11).

The usefulness of BIS monitoring during general anesthesia has been widely validated in multiple adult and pediatric studies (1,11,12,15,17-20). However, few studies have focused on the particularities of dental treatment in patients with mental disorders.

Thus, the aim of the present study was to compare the observed changes in heart rate (HR), oxygen saturation (SaO2), respiratory rate (RR), endtidal carbon dioxide (EtCO2) and blood pressure (BP) after the administration of propofol and sevoflurane in children with special needs (CSN) versus healthy children (HC) during dental treatment, with the use of BIS monitoring.

## Material and Methods

-Sample description

The present prospective, non-randomized consecutive clinical trial was approved by the Ethics Committee of the Faculty of dentistry of the University of Murcia (Murcia, Spain) (Committee registry number 1459-2017).

All uncooperative pediatric patients requiring dental treatment in a private clinic in Cartagena (Spain) were considered eligible during the period between September 2019 and March 2020. The inclusion criteria were: a) patient age 2-16 years; b) ASA score I, II or III; c) need for dental treatment (extractions, restorations, pulp therapy, etc.); and d) impossibility to perform treatment under local anesthesia. The exclusion criteria were: a) patients under 2 years or over 16 years of age; and b) ASA score IV.

The study sample consisted of 40 children, considering a previous sample size calculation of a minimum of 17 individuals per group (with 95% confidence and an accuracy of ± 5%). The patients were allocated to two groups: 1) a control group of healthy children (HC); and 2) a study group of children with special needs (CSN) according to the classification of Maeda *et al*. (16) regarding function, disability and health in relation to the tolerability of dental treatment.

Informed consent was obtained prior to the investigation from all parents after fully explaining the benefits, inconveniences and potential risks of the intervention.

-Treatment management

The patients received no anxiolytic premedication before the treatment day. At baseline and prior to the administration of any other drugs, we started continuous monitoring of vital signs with a Datex-Ohmeda Type F-FM Monitor (Instrumentarium Corp., Helsinki, Finland). A pediatric blood pressure cuff was placed on the right arm; a pulsioxymetry probe was attached to the index finger of the left hand; and three electrodes for continuous ECG monitoring were placed. In all cases, BIS monitoring was performed using a Covidien BIS Complete Monitoring System® (Covidien Inc., Mansfield, MA, USA) employing commercially available pediatric BIS sensor strips. Using an algorithm for digital signal processing, a numerical value known as the BIS index was obtained, ranging from 0 to 100, where 0 represents no brain activity (seen in coma and brain death) and 100 indicates an awake patient (3,11,15) (Table 1). The skin was cleaned with ethyl alcohol before the BIS sensor was placed, pressing lightly on the skin for 5 seconds to ensure adhesive contact between the skin and the sensor for obtaining good signal quality. The pediatric sensor was placed on the patient forehead and temporal region above the zygomatic arch using self-adhesive. All the monitoring parameters were continuously displayed and recorded during the entire dental treatment procedure.

**Table 1:**
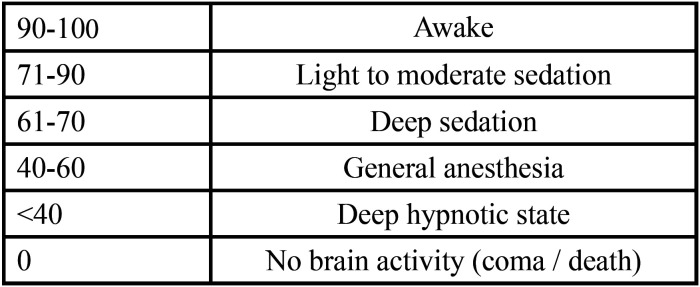
The BIS index.

Then, 2% sevoflurane in oxygen was administered on a continuous basis (Mapleson ventilation system, 100% oxygen, 5 l/min). When the BIS values were between 60-70, a venous access was prepared on the back of the right hand using a 22G catheter, with the start of continuous propofol infusion (Target-Controlled Infusion, TCI®; Alaris PK Care Fusion, UK) to obtain a targeting effect compartment concentration of 2 µg/ml in two minutes. At the same time, we started to decrease the inhaled sevoflurane concentration to 1%.

A flexometallic laryngeal mask (LMA, Proseal® airway, Teleflex Medical Europe, Ltd.) (especially designed to isolate the airway from the digestive tract and prevent pulmonary aspiration) of the required number according to the weight of the patient was inserted and fixed on the side opposite to the mouth-opener for continuous oxygen administration (2 lpm), under spontaneous breathing conditions at all times.

Once the anesthetist indicated that optimum sedation had been achieved and was maintained with propofol 2 μg/ml and 1-2% sevoflurane, with the BIS value between 40-60, the dentist began the administration of local anesthesia (2% lidocaine with 1:80,000 adrenaline). The amount of local anesthesia used was recorded. The patients remained in a nearly supine position during the dental procedure. Data were recorded at baseline, the start of treatment, and every 15 minutes until the end of the treatment procedure.

Dental treatments were performed using the usual techniques and were recorded for each patient. In those cases where treatment was expected to last long, dexamethasone 0.2-1 mg as an antiinflammatory and antiemetic measure was administered. Atropine 0.01 mg/kg was also administered in order to avoid excess mucous secretion, if required.

In the event of patient discomfort caused by the treatment stimulus (evidenced by movement, complaint, increased HR and/or BP or increased level of consciousness), the anesthetist raised the sevoflurane concentration to 3% for 1-2 minutes and incremented propofol to 2.5-3 µg/ml. We also recorded any unexpected events, such as a need for ventilation support. In such situations the anesthetist first checked the position of the LMA and set the mandible in hyperextension in order to prevent tube bending and facilitate oxygen entry to the airway.

At the end of dental treatment, propofol and sevoflurane were stopped. When the patient presented a BIS value of 80-90, the LMA was removed and spontaneous breathing was maintained with manual help according to the individual wake-up time. Then, the patient was moved to the anesthesia recovery room with the parents. All patients were kept under observation for 30-45 minutes after surgery, with pulsioxymetry and blood pressure monitoring. They were then discharged in accordance with the usual criteria for ambulatory major anesthesia: stable vital signs, the ability to stand, no bleeding, no nausea and/or vomiting, and no moderate or severe pain.

Postoperative instructions were provided, and analgesic (dexketoprofen) and antiemetic medication (metoclopramide hydrochloride) was prescribed. The parents were called by phone on the same day in the afternoon and again on the following day to assess possible complications.

-Statistical analysis

The monitoring data collected during the entire procedure were blinded to the main investigator, and a single investigator assessed the level of alertness and recorded all the data in order to eliminate interobserver variability.

Variables such as gender, age, weight, the need for chronic medication, presurgical medication, dental treatment (restorations, pulpotomies, extractions, sealing, topical application of fluoride, tartrectomy, root treatment or the placement of preformed crowns), intravenous sedation drugs and lidocaine carpules were recorded for each group, with the mean and standard deviation (SD).

To study the variation over time of the variables in both groups, two-factor analysis of variance (ANOVA) and the Pearson correlation coefficient were used, with repeated measures to check the effects of time and group, and the interactions between them. A 95% confidence level was considered, with statistical significance being accepted for *p* < 0.05. The Statistical Package for the Social Sciences version 25.0 (SPSS® Inc., Chicago, IL, USA) for MS Windows was used throughout.

## Results

-Sample description

The study sample of 40 patients comprised children between 2 and 13 years of age. The control group consisted of 19 healthy children (9 males and 10 females), and the study group consisted of 21 patients (13 males and 8 females) presenting the following mental disorders: 7 with autism spectrum disorder (one of them also with Asperger’s syndrome), 5 with Down syndrome, four with chronic encephalopathy, one with Cayler syndrome, one with Turner syndrome, one with cerebral palsy, one with hydrocephalus and one with language disorder.

The CSN group was significantly older than the HC group (mean 8 ± 2.7 versus 5.5 ± 1.8 years; *p*=0.001). Nevertheless, the groups were homogeneous in terms of gender, body weight, chronic medication, presurgical medication and drugs administered during the treatment provided in the present study (*p*>0.05). The descriptive and comparative analysis of the drug doses showed that the patients in both groups received similar intravenous agents (propofol, sevoflurane), with no significant differences between them (*p*<0.05). Likewise, no significant differences were observed in terms of the local anesthesia administered in both groups (*p*<0.05) (Table 2).

**Table 2:**
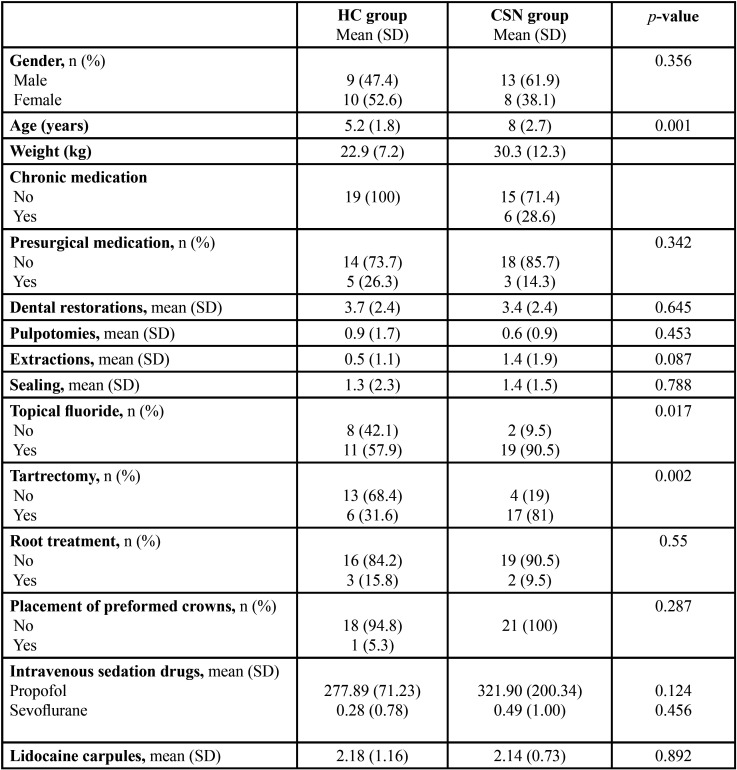
Demographic data, patient treatment and intravenous sedation drugs employed.

-Dental treatment description

Dental treatments comprised the topical application of fluoride, tartrectomy, restorations, pulp therapies and extractions, with the use of local anesthesia when needed. No statistically significant differences (*p*>0.05) between the two groups were observed in terms of the dental treatments provided, with the exception of the topical application of fluoride (*p*=0.017) and tartrectomy (*p*=0.002). Topical fluoride application was performed in 90.5% of the patients in the CSN group, and in 57.7% of the patients in the HC group. Similarly, tartrectomy was performed in 81% of the patients of the CSN group, versus in 31.6% of the patients in the HC group. All these data are fully described in Table 2.

-Hemodynamic and ventilatory parameters

A significant decrease in systolic blood pressure (SBP), diastolic blood pressure (DBP) and RR was observed over time (*p* <0.001), though no statistically significant differences were observed between the two groups for any of these variables.

**Figure 1:**
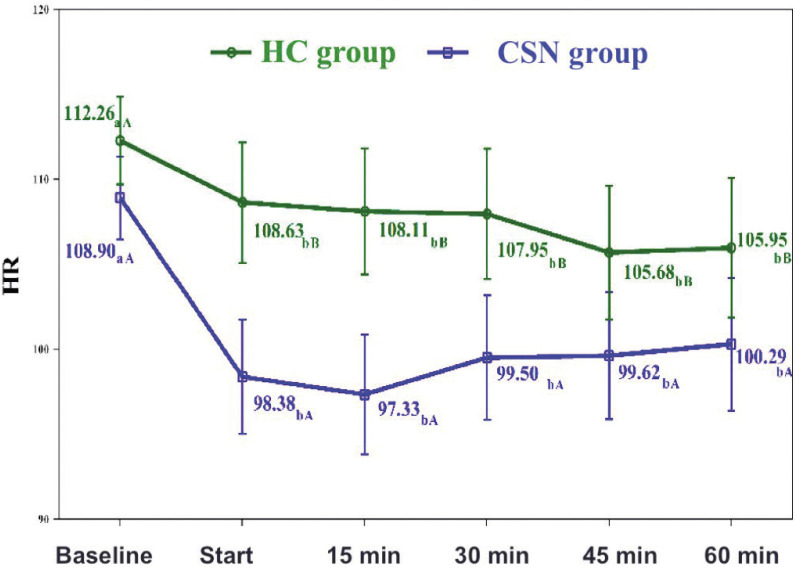
Heart rate. Group and time interaction. a-d. Two-by-two comparisons. In the same group, different lowercase letters indicate statistical-ly significant differences between the timepoints (Bonferroni correction). A-B. Two-by-two comparisons. At the same timepoint, different capital letters indicate statistically significant differences between the groups (Bonferroni correction).

Heart rate also decreased significantly over time. In both groups, HR decreased significantly at the treatment starting timepoint versus the baseline value, and remained without statistically significant changes from that moment until the end of the study. However, there was also a significant interaction effect between both groups over time. The mean HR from the start of the intervention until the end of treatment was significantly lower in the CSN group (100.67 bpm) than in the HC group (108.09 bpm) (*p* = 0.012) (Fig. 1).

Lastly, in relation to the variables SaO2 and expired CO2, no statistically significant differences were observed over time and no interactions between the two groups were recorded.

-Bispectral monitoring

**Figure 2:**
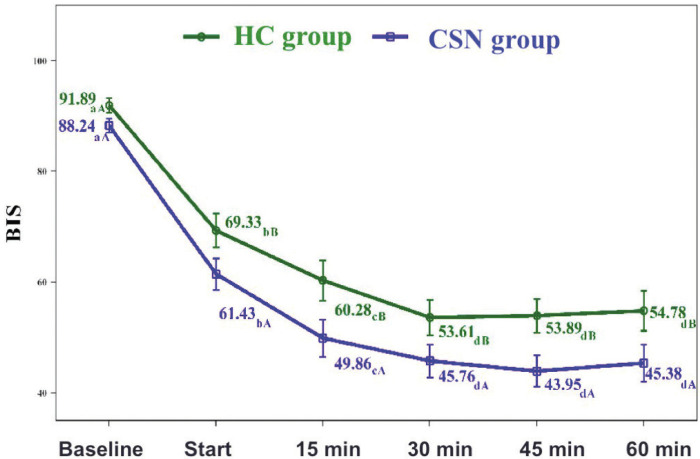
BIS value. Group and time interaction. a-d. Two-by-two comparisons. In the same group, different lowercase letters indicate statistically significant differences between the time points (Bonferroni correction). A-B. Two-by-two comparisons. At the same timepoint, different capital letters indicate statistically significant differences between the groups (Bonferroni correction).

The BIS values also decreased significantly over time in both groups. Likewise, there was a significant interaction effect between both groups. In this case, there was a statistically significant decrease from the start of treatment until the 30-minute timepoint. From this moment until the end of the study, there were no statistically significant changes in either of the two groups. How-ever, from the start of the intervention, the BIS values in the CSN group were significantly lower than in the HC group (*p* = 0.043) (Table 3, Fig. 2). The mean BIS value in the CSN group was 55.77 versus 63.06 in the HC group. The lowest mean BIS value was 43.95, and was recorded at minute 45 in the CSN group.

**Table 3:**
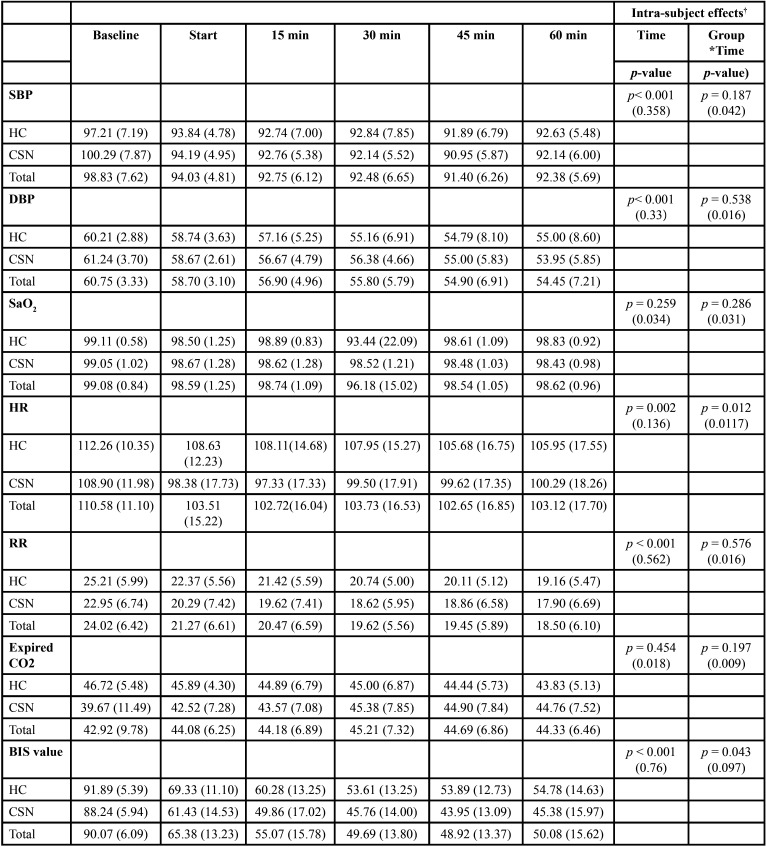
Variation of the study variables over time.

The variation over time of the hemodynamic and ventilatory parameters (systolic and diastolic BP, SaO2, HR, RR, CO2) and the BIS values between the two groups is summarized in Table 3. No intra- or postoperative complications were recorded.

## Discussion

In both healthy children and pediatric patients with mental disorders, dental anxiety is a prevalent phenomenon that can complicate the provision of dental treatment under appropriate safety standards (4,13). An increase in BP and HR can be expected during dental treatment, and this can result in altered cardiac rhythm, angina or myocardial infarction in patients with established car-diovascular disorders - though such complications can also be found in healthy patients (13). Therefore, a good and effective alternative in the case of pediatric patients is to perform dental treatment under intravenous sedation or general anesthesia in order to reduce dental anxiety, provide a comforTable and reliable surgical environment (21) and prevent stress-related complications during dental treatment (3).

General anesthesia can assist in providing quality dental care in many patients who could not be treated otherwise. This is especially true for children with special needs who increasingly attend dental clinics for treatment (8). In this regard, the American Academy of Pediatric Dentistry defines children with special health care needs as those who have “any physical, developmental, mental, sensory, behavioral, cognitive or emotional impairment or limiting condition that requires medical management, health care intervention, and/or the use of specialized services or programs“ (10). The most common disease in the CSN group in our study was autism, followed by Down syndrome and chronic encephalopathy. Although some authors comment that cerebral palsy (22), autism (23,24), intellectual impairment (24) or encephalopathy (25) are the most prevalent disorders in children with special needs subjected to general anesthesia, other authors comment that no relationship has been found between systemic disease and the use of general anesthesia (8).

In our study, the mean HR and BIS values were lower during treatment in the CSN group compared to the HC group. This is in contradiction with the observations of Malhotra *et al*. (14), who found no significant differences in hemodynamic parameters on comparing midazolam/ketamine in combination versus dexmedetomidine (14). These discrepancies may be due to multiple causes, such as different depths of sedation, the different drugs employed, the younger age of the patients in the study published by Malhotra *et al*. (14) (3-6 years), or differences between the healthy pediatric patients in their study and our own study group in which the patients had mental disorders that could be accompanied by other systemic conditions capable of influencing HR and BIS. In this regard, it must be remembered that cardiac alterations are found in some of the syndromes present in the CSN group (Down syndrome, Cayler syndrome and Turner syndrome).

The variables BP, RR, SaO2 and EtCO2 behaved slightly differently in the two groups, but there were no statistically significant differences over treatment time. Saravia *et al*. (26) did not measure CO2, but recorded fluctuations in HR and systolic and diastolic BP during treatment under sedation. They associated these changes to environmental stimuli, though no significant variations were observed between the preoperative and intraoperative recordings. The mentioned authors found 41% of the sedated patients to develop mild hypoxia and 6% moderate hypoxia during sedation (26); this is in contrast to the findings of our own study, where none of the patients presented hypoxia episodes.

The lowest BIS values observed in the present study corresponded to the CSN group, employing the same dose of anesthetic drug. It could be postulated that the age of the patient might influence the BIS data (12,27), as observed by Bannister *et al*. (28), who assessed the effect of the anesthetic upon the BIS values in children under general anesthesia, affirming that the results differ according to the age of the patient group involved.

Previous studies have found the BIS pattern in cerebral palsy (27) and in mentally retarded children (autism, cerebral palsy and Down syndrome) (29) to follow the same trend as in normal patients during general anesthesia in dentistry. This is in contrast to our own findings, since the BIS scores in the CSN group were significantly lower than in the HC group. Our results are in concordance with those reported by Choudhry and Brenn (27). Although these authors concluded that BIS monitoring in children with cerebral palsy showed a pattern of change similar to that observed in normal children, the absolute BIS values were lower than those recorded in the normal children at four of six measurements timepoints (27). Likewise, Valkenburg *et al*. (30) found the BIS values to be significantly lower in intellectually disabled children compared with controls in percutaneous endoscopic gastrostomy performed under general anesthesia. According to the mentioned authors, these lower values possibly could be explained by the fact that some brain disorders may exhibit epileptiform or non-epileptiform forms, or need anticonvulsant medication.

In line with other investigations, bispectral monitoring was used efficiently and effectively in mentally retarded patients (31,32), and could have a positive effect upon the recovery profile in developmentally delayed pediatric patients (32).

Although general anesthesia is a safe procedure, postoperative dental morbidity or complications have been described (25). No intra- or postoperative complications were recorded in our study.

In the study published by Pecci-Lloret (25), no postoperative medical or dental complications were observed, and the patients were discharged 12-15 h after the intervention. In the same way, Caputo *et al*. (33) found the incidence of mortality to be minimal, and morbidity was limited to minor events in this group of patients. Thus, based on the data found in the literature, it can be affirmed that general anesthesia is a safe and successful procedure for performing dental treatments in children with special needs, and parental satisfaction and acceptance of dental treatment under general anesthesia has progressively increased in recent years (25).

Lastly, some questions and limitations arise from our study. Although the literature on the use of BIS in dentistry is increasing, the majority of studies have been conducted in adults. In this regard, data referred to adults might not be applicable to the pediatric population (27). Furthermore, the literature offers less information about the validity of BIS monitoring in the case of children with systemic diseases or with neuronal disorders, since a low value could be attributed to brain disease or to medication taken by the patient (e.g., anticonvulsants). Apart from this, the small number of individuals included in our study may appear to be a clear limitation. It is difficult to recruit a sufficient number of subjects corresponding to vulnerable populations such as children with mental disorders. Nevertheless, the number of patients recruited was sufficient to find statistically significant differences.

To meet future expectations, attempts should be made to match the two study groups so that age shows no statistically significant differences, and determine whether neuronal maturation affects the BIS values. Homogenizing mental diseases within the sample as far as possible, and taking into account that other systemic diseases may be present in certain mental syndromes, would facilitate the evaluation of their impact upon the BIS values and vital signs.

## Conclusions

In conclusion, propofol and sevoflurane administration allows dental treatment to be performed safely in children with special needs who otherwise would not be treated. These drugs cause a significant decrease in heart rate and bispectral values in children with special needs versus healthy children. Blood pressure, oxygen saturation and exhaled carbon dioxide showed similar results in both groups.
